# Nutrition and Food Literacy in the MENA Region: A Review to Inform Nutrition Research and Policy Makers

**DOI:** 10.3390/ijerph191610190

**Published:** 2022-08-17

**Authors:** Hala Mohsen, Yonna Sacre, Lara Hanna-Wakim, Maha Hoteit

**Affiliations:** 1 Doctoral School of Sciences and Technology (DSST), Lebanese University, Hadath 1103, Lebanon; 2 Faculty of Public Health, Section 1, Lebanese University, P.O. Box 6573, Beirut 1105, Lebanon; 3 PHENOL Research Group (Public HEalth Nutrition prOgram Lebanon), Faculty of Public Health, Lebanese University, P.O. Box 6573, Beirut 1105, Lebanon; 4 Lebanese University Nutrition Surveillance Center (LUNSC), Lebanese Food Drugs and Chemical Administrations, Lebanese University, P.O. Box 6573, Beirut 1105, Lebanon; 5 Department of Nutrition and Food Sciences, Faculty of Arts and Sciences, Holy Spirit University of Kaslik (USEK), P.O. Box 446, Jounieh 1200, Lebanon; 6 Department of Agricultural and Food Engineering, School of Engineering, Holy Spirit University of Kaslik (USEK), P.O. Box 446, Jounieh 1200, Lebanon

**Keywords:** food literacy, nutrition literacy, MENA, malnutrition, food insecurity, researchers, policymakers

## Abstract

Background: Improving food and nutrition literacy is fundamental to tackling the Middle East and North Africa (MENA) region’s enormous challenges, including malnutrition and food insecurity. To direct initiatives, it is crucial to assess the region’s food and nutrition literacy. Thus, we aimed to review studies on food/nutrition literacy status in the MENA countries and illuminate the region’s research gaps in these areas, in terms of assessment, policy, and program implementation. Methods: PubMed and Google Scholar databases were searched between 18 December and 8 May 2022, to identify relevant articles published up to 2022 in the MENA region. Results: Twelve studies were included in this review. Lebanon, Palestine, and Iran are the only three MENA countries where nutrition and/or food literacy were assessed. People in these countries mostly had inadequate food and/or nutrition literacy levels, especially in the skills rather than the cognitive domain. Food and/or nutrition literacy showed associations with food habits, food-label use, food-consumption patterns, school performance, food security, dietary diversity, and nutrient adequacy. The MENA countries developed no policies or programs to address food and nutrition literacy. Conclusion: This review is a wake-up call for researchers and policymakers to develop a robust approach to combat food and nutrition literacy concerns in the MENA region.

## 1. Introduction

### 1.1. Malnutrition and Food Insecurity Burden in the MENA Region

The triple burden of malnutrition and anemia along with the coexistence of food insecurity are tremendous challenges in the MENA region [1]. With ongoing conflicts, political instability, and the COVID-19 pandemic, the region is witnessing an unprecedented increase in food insecurity, a decline in dietary diversity [2], the adoption of unhealthy eating patterns, and changes in food literacy [3]. In addition, the ongoing conflict in Ukraine, since February 2022, is compounding the impacts of two long years of the COVID-19 pandemic on economies, food insecurity, poverty, and malnutrition in the MENA region [4]. It is expected that the number of people affected by hunger in the region will surpass 75 million by 2030 [5]. Recent estimates show that more than 7 million children in the region suffer from chronic malnutrition, manifested as stunting [6]. Besides, 3.7 million children have acute malnutrition, of which 1.6 million are severely wasted [6]. The average prevalence of overweight and obesity was 27% and 24% in adults and 16.5% and 4.8% in school-aged children, respectively [7]. Besides, anemia prevalence in the region ranged between 22.6% and 63% amongst pregnant women, 27% and 69.6% amongst women of reproductive age, and 23.8% and 83.5% amongst under-five children [8]. Added to these, MENA’s share of the world’s acutely food-insecure people was 20%, which is considered high for its 6% share of the population [9]. Hence, to overcome these challenges, the MENA countries should re-prioritize and reform inefficacious practices and food-related policies towards “high-return nutrition investment” that amplifies the availability of and the access to nutritious foods [10]. Furthermore, the reform of incompetent food-related practices and policies offers resources that can be used to ramp-up well-targeted educational, social, behavioral, nutritional, and health-related outcomes [11]. These outcomes are needed to transform and enhance the resilience of the region’s food systems, to increase the capacity to ensure safe, healthy, and sustainable diets [11]. To achieve Zero Hunger by 2030, it is crucial to transform Arab food systems to end hunger and malnutrition [11]. Thus, some various elements should be the focus of intervention. These include nutrition knowledge and attitudes, food and cooking skills, food environment, food preferences, and food literacy [12]. Being food literate is crucial to help people make healthy food choices that ensure nutritional needs are met [13]. “Food literacy is particularly important in the early years, when children are developing the eating patterns and skills that they will carry into adulthood and pass on to future generations” [13].

### 1.2. Existing Definitions of Food Literacy and Nutrition Literacy

The term “food literacy” has gained momentum in recent years, being evolved from the broader concept of health literacy [14]. Researchers will continue to debate the definition of food literacy for many years until it becomes a rich study area [14]. Food literacy is defined as the ability to make appropriate food decisions to support individual health and a sustainable food system, considering environmental, social, economic, cultural, and political contexts [15]. Food and nutrition knowledge, food skills, self-efficacy and confidence, food decisions, and multiple environmental factors, particularly the food system, are all food literacy attributes, according to the Food Literacy Framework for Healthy Eating [16]. Widener, P. and Karides, M. (2014) [17] further suggested the term “system food literacy”, implying that food literacy entails a thorough understanding of the food system and its social and economic issues. Besides, the components of functional (comprehend and understand information), interactive (share and exchange information and interact with others), and critical (evaluate and judge information) literacy presented by Nutbeam’s tripartite model [18] are reflected in worldwide food-literacy definitions.

Similar to food literacy, nutrition literacy is under the umbrella of health literacy [14]. It is considered “a health literacy applied to the field of nutrition” [14]. The nutritionally literate individual is one who can obtain, process, and understand basic nutrition information necessary for making appropriate nutrition decisions [19]; however, food literacy is incorporated into a broader spectrum of theoretical and practical knowledge and skills [20]. In other words, the skills incorporated in nutrition literacy are prerequisites for food-literacy competencies [20]. Nevertheless, they do not represent the whole spectrum of skills and competencies needed to perform well in making healthy and right food decisions [20]. Food and nutrition literacy are distinct but complementary concepts, as they are not debated as freestanding terms [20]. Food-literacy and nutrition-literacy definitions have not been originated by Arab authors yet, indicating that these topics are undervalued in the region. The definitions of food literacy and nutrition literacy over the years are summarized in Table 1.

### 1.3. Food and Nutrition Literacy and Nutrition Outcomes

Promoting food and nutrition literacy is a determinant factor in leading to healthy food choices and the adoption of healthy diets by children and adolescents, in particular [28]. Plenty of evidence shows that adequate levels of food and nutrition literacy are positively associated with food selection, food preparation, eating habits, and diet quality [28]. For instance, a higher level of food and nutrition literacy has been associated with increased consumption of fruits and vegetables [29], preference for healthy food [30], and decreased consumption of prepacked or processed food [31]. Besides, having limited food skills was shown to be associated with increased consumption of ultra-processed foods [32]. Above these, inadequate food literacy had exacerbated food insecurity by impeding adequate food utilization [33]. Though evidence demonstrating the relationship between nutrition outcomes and food and nutrition literacy is still developing, the foregoing findings suggest that food- and nutrition-literacy-based interventions in the MENA region are extremely promising.

### 1.4. Rationale of the Study and Study Objectives

As outlined above, in order to address the region’s emerging challenges, it is necessary to adopt a package of intervention measures, focused on improving food and nutrition literacy and guided by a thorough analysis of the region’s nutrition situation, especially in the critical stages of life, such as childhood and adolescence. In fact, inadequate nutrition in childhood and adolescence can potentially retard growth [34]. Equipping people with adequate nutrition knowledge and food skills is one promising approach to reducing MENA’s malnutrition and food-insecurity burden [35,36,37]. In light of this, and in order to direct initiatives, additional information about the state of food literacy and nutrition literacy in the MENA region is needed. Therefore, in this paper, we aimed to:

1. Review studies on the food-literacy and nutrition-literacy status in the MENA countries.
2. Illuminate the region’s current research gaps in these areas, in terms of assessment, policy, and program implementation.

## 2. Methods

### Search Strategy and Data Extraction

**Figure 1:**
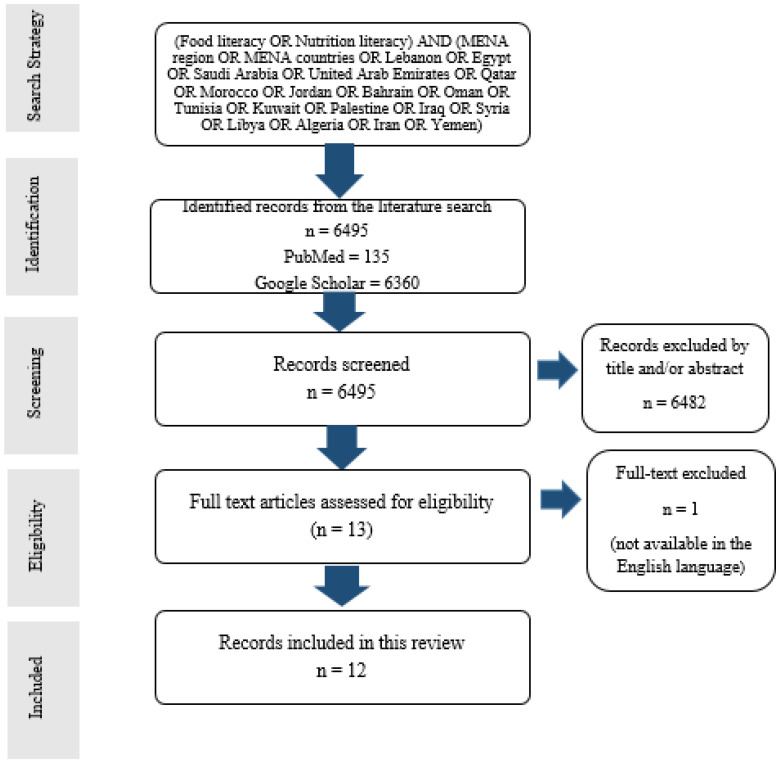
The flow diagram of the search and identification process for the 12 studies included in this review.

We conducted an extensive review of the literature on studies on the assessment of food literacy and nutrition literacy in the MENA countries published up to 2022 using the online databases: PubMed and Google Scholar. We also hand-searched for the relevant literature using snowballing by accessing reference lists to identify additional papers. The following search terms were used: “food literacy”; “nutrition literacy”; “MENA region”; “MENA countries” “Lebanon”; “Egypt”; “Saudi Arabia”; “United Arab Emirates”; “Qatar”; “Morocco”; “Jordan”; “Bahrain”; “Oman”; “Tunisia”; “Kuwait”; “Palestine”; “Iraq”; “Syria”; “Libya”;” Algeria”; “Iran”; “Yemen”. These keywords were chosen by referring to the latest classification of the MENA countries according to the Office of the United States Trade Representative (USTR) [38], which are presently the 18 countries listed above. The keywords were used in various combinations to find relevant articles. This search was performed between 18 December and 8 May 2022. Filters and limits included (in PubMed database) the English language and the human species to avoid irrelevant searches. The reviewers screened the title and abstract of the citations to highlight the suitable articles relevant to the objective of the review for further inclusion. Eligible studies included those that assessed food literacy and/or nutrition literacy in any MENA country, used a cross-sectional design, and included healthy participants of any age. For those articles that appeared relevant, the full-text study report was obtained and data were extracted, to be documented in an Excel spreadsheet using a prepared template including the following information: study title, authors’ names, year of publication, country, study objectives, sample size, age group, study instruments, and relevant findings. The detailed search strategy and identification process of included articles are shown in Figure 1.

The review findings on the status of food and/or nutrition literacy were compared with that obtained outside the MENA region to allow for comparison and discussion. Besides, we reviewed food literacy and nutrition literacy policies and programs with the countries of their implementation, to identify if any have been implemented in the MENA region.

## 3. Results and Discussion

Overall, 12 studies were included in this review. Table 2 presents the data extracted from each study. Among them, five were published in the year 2021 [39,40,41,42,43]; four in 2020 [33,44,45,46]; one in 2019 [47]; and two in 2018 [48,49]. Regarding the country of origin of the studies, one study was conducted in Lebanon [39], two studies were conducted in Palestine [40,41], and the nine remaining were performed in Iran [33,42,43,44,45,46,47,48,49]. In addition, eight of the studies were assessing food and/or nutrition literacy among adolescents [33,39,42,43,44,45,47,49], while the remaining studies included adult participants [40,41,46,48]. Adolescents were considered as individuals aged between 10–19 years old, according to the recommended criteria of the World Health Organization (WHO) [50]. Besides, the sample size in the reviewed studies ranged between 101 and 803 participants. All in all, five studies assessed both the food literacy and nutrition literacy of the participants [33,42,43,44,47], whereas the remaining seven studies assessed nutrition literacy alone [39,40,41,45,46,48,49]. However, no studies solely addressed the food-literacy concept.

### 3.1. Assessment Tools

To assess food and nutrition literacy in the MENA countries, the Food and Nutrition Literacy Assessment Tool (FNLAT) and the Food and Nutrition Literacy Tool (FNLIT) were used on the reviewed studies. FNLAT was used on only one study [42], while FNLIT was used on five studies [33,43,44,45,47]. For nutrition-literacy assessment, six different tools were used: the Nutrition Literacy Assessment Instrument (NLAI) [39]; Newest Vital Sign (NVS) [40]; Nutrition Literacy Scale [41]; NLQ-20 [49]; Nutrition Literacy Instrument, developed in Turkey [48]; Evaluation Instrument of Nutrition Literacy on Adults (EINLA) [46].

Multiple tools with different psychometric properties also exist and were used outside the MENA boundaries; these include the Food Literacy Assessment Tool for Healthy, Joyful, and Sustainable Diet in South Korea [51], Food and Nutrition Literacy Questionnaire for Chinese School-age Children (FNLQ-SC) [52], a food literacy instrument for school children in a Danish context [53], Food Literacy Assessment Tool (FLitT) in the United States (U.S.) [54], Thai-Nutrition Literacy Assessment Tool for Adolescents (Thailand) [55], preschool-FLAT for Italian children [56], Tool for Food Literacy Assessment in Children (TFLAC) in U.S. [57], Critical Nutrition Literacy Scale (CNL-E) (Norway) [58], Menu Board Literacy, and Self-Efficacy Scale for Children in the U.S. [59], the dietary behavior scale and the self-efficacy in science scale (Norway) [60], Food Label Literacy for Applied Nutrition Knowledge (FLLANK) Questionnaire in the U.S. [61], Adolescent Nutrition Literacy Scale (ANLS) in Turkey [62,63,64,65], 19-item food literacy measurement tool in Korea [66], Nutrition Literacy Scale-Greek (NLS-Gr) [67], Your PEL—Promote and Empower for Health Literacy (with a food-literacy scale) in Portugal [68], NLit-P in the U.S. [69], Spanish Nutrition Literacy Scale [70], Electronic-Nutrition Literacy Tool (eNutLit) [71], Short Food Literacy Questionnaire (SFLQ) [72], and Italian Food Literacy Survey (IT-FLS) [73].

### 3.2. Food and/or Nutrition-Literacy Status in the MENA Countries

Taleb, S. and Itani, L. (2021) [39] assessed the nutrition literacy among 189 Lebanese adolescents aged 14–19 years old and the association of nutrition literacy with adolescents’ BMI status and food habits. Lebanese adolescents had adequate nutrition literacy on the nutrition and health, macronutrients, and food-groups literacy components of the NLAI scale; however, marginal nutrition literacy was observed for household-food measures and food-label reading [39]. In contrast, Natour, N. et al. (2021) [40] reported that about one quarter (29%) of Palestinian adult participants appeared to have adequate nutrition literacy. Tell et al. (2021) [41] further found that the mean of functional nutrition literacy (FNL) was 2.8 ± 0.5 (over 7), 3.3 ± 0.5 (over 8) for interactive nutrition literacy (INL), and 3.6 ± 0.5 (over 11) for critical nutrition literacy (CNL), indicating literacy insufficiency at all sublevels among Palestinian adults.

The food and nutrition literacy has been widely addressed in Iran more than any other country in the MENA region. Ashoori, M. et al. (2021) [42] explored that Iranian senior-high-school students (17–18 years old) had inadequate levels of food and nutrition literacy, with a mean ± SD of the total food and nutrition literacy (FNL) score equal to 52.1 ± 10.96 (a score of 60 is considered acceptable) [42]. Among 803 Iranian student participants (10–12 years old), 68.8% of them had high food and nutrition scores in the cognitive domains (understanding food and nutrition information); nevertheless, at least one out of four students (25%) had low score values in the skill domain (including food label literacy, food choice literacy) [43]. Moreover, the study findings by Doustmohammadian et al. (2020) [44] reemphasized the latter, by showing that 25% of school-age Iranian students had low scores in the skill domain, and the majority (97.4%) scored moderate-to-high in the cognitive domain of the food and nutrition literacy. Similar findings were also reported in a 2019 published paper [47], which explored that even though most Iranian adolescents had adequate food and nutrition literacy, more than half (69%) had the highest score in the cognitive domain, while only few (15%) scored highly in the skills domain. Khorramrouz, F. et al. (2020) [33] demonstrated that 14% of 315 Iranian students had inadequate food and nutrition literacy, and only 23.2% had high food- and nutrition-literacy scores. Mehri et al. (2020) [45] also reported that most Iranian adolescents had poor food and nutrition literacy (62% of males and 58.1% of females). As well, the nutrition literacy score in elementary school teachers in Yasuj, Iran was 27.14 ± 3.2, revealing that 22.7% of participants were nutritionally illiterate [48]. Added to these, the mean ± SD of total nutrition literacy was 52.98 ± 7.15, an indication of inadequacy in literacy levels among Iranian young adolescents (13–15 years old) [49]. On the contrary, about half of Iranian medical students (48.1%) showed adequate nutrition literacy, and, interestingly, only 1% had poor nutrition-literacy scores [46].

Therefore, food literacy and nutrition literacy have been inadequately addressed in the MENA countries, with the most data available in Iran. Moreover, where assessed, the data seem distressing, with critical deficits in skills rather than cognitive areas, such as food-label reading, food selection, and cooking. Nonetheless, food literacy and nutrition literacy were shown to be of better status in multiple countries outside the MENA region, even among adolescent participants. This was evident in recent studies conducted in China [74], Turkey [62,63,64], the U.S. [75], and Ghana [76], with all emphasizing adequate nutrition literacy in the sampled population, showing good competencies in most or even all food-literacy and nutrition-literacy components. Besides, good nutrition-literacy levels and basic food literacy were recorded in Greece [67] and Canada [77], respectively. The disparity between the MENA countries and other nations’ findings may be attributed by the latter’s investment in food and nutrition education. For instance, in the U.S., the percentage of schools providing nutrition education and instructions was 74.1% in 2014 [78]. Besides, it is encouraging to note that Nigeria has decided to increase investment in school food and nutrition [79]. Evidence suggests that people can change their behaviors to improve their nutrition outcomes, particularly when they are in supportive environments [80]. Thus, in a region such as the MENA that is plagued by numerous conflicts, the people, especially children and youth, need opportunities to develop nutrition and food literacy through food and nutrition education, standards, and policies. In addition to national governments, activists and advocates have a role to play in bringing about change. This is in line with the call for systemic change made by Agenda 2030 and the emphasis placed on food systems in the outcome documents of the Second International Conference on Nutrition (ICN2).

Most of the reviewed studies in the MENA region included adolescent participants, with all reporting dismal literacy levels in this vulnerable age group. This observation is crucial because adolescence encompasses critical stages of development and rapid growth, mainly the pubertal growth stage, during which nutrient requirements increase [79]. Accordingly, nutrition-related problems have been observed among adolescents having inadequate levels of nutrition literacy [13]. Therefore, it is essential to promote healthy eating habits during this unique life stage, by having interventions be delivered continuously, with a specific emphasis on school-based food and nutrition education. Besides, the review presently reveals that skill-based literacy, mainly food-label use, is more problematic than cognitive-based literacy, with lower use of food labels among food/nutrition-illiterate participants [40,42,43,44,47]. Based on many pieces of evidence, food-label reading is one pivotal step to improving food choices and dietary habits [81]. One possible explanation for observing such findings in the MENA countries is that this concept and its application have neither been entered into school curricula and textbooks nor in education programs, to empower individuals in basing their food choices according to nutrition- and safety-related considerations. One recent study showed that Lebanese shoppers expressed poor nutrition-label-related knowledge, and more than half the population either read nutrition labels occasionally or did not look at the food label at all [82]. Emirati consumers reported looking mostly at the expiration date of the products rather than the information related to the food-storage and -handling instructions and the biotechnology as well, which are key components of being food literate [83]. Similarly, only 42% of Bahraini consumers read the food label, with the common practice being reading the basic label information related to production and expiry dates [84]. Food-literacy interventions focusing on consumer’ skills to read food labels should be prioritized in such regions, where nutrition inadequacy and malnutrition are not uncommon. Thereupon, Hoteit M. and colleagues (2022) [82] provided data on this topic, “the first steps in the Nutri-Score roadmap”, to serve as an initiative to motivate the national implementation of food-labeling approaches in Lebanon such as the Nutri-Score front-of-pack label.

### 3.3. Correlates of Food and/or Nutrition Literacy in the MENA Countries

This review found associations between food and/or nutrition literacy and critical factors and correlates. The nutrition literacy of Lebanese adolescents was associated with their food habits; nevertheless, an unusual finding is that participants with higher nutrition literacy scores had poorer food habits (higher fat intake, higher sweets intake, and lower intake of fruits and vegetables) as compared to other participants with low nutrition-literacy scores [39]. Related to this, Taleb, S. and Itani, L. debated that nutrition literacy alone, if not coupled with essential behavior capabilities and environmental support, may not guarantee behavior change [39]. What is also worth mentioning is that the body mass index (BMI) of Lebanese adolescents did not correlate with their nutrition literacy [39]. However, higher nutrition-literacy levels among Palestinian adults predicted better use of the food labels as well as checking the health benefits on the label and, in consequence, higher consumption of low-calorie products [40]. In addition, the food and nutrition literacy were correlated with the use of food labels and looking, in particular, over the ingredients part of the label [41]. Adding cheese and mayonnaise to meals was less common in participants with adequate critical food and nutrition literacy [41]. Participants with better FNL seemed to rely on health professionals and scientific books rather than unreliable online sources to seek nutrition information [41].

Ashoori, M. et al. [42] found that high food and nutrition literacy scores predicted better school performance among high school students. Participants who had a family member having a nutrition-related disease scored higher on the food-label-reading skill of the food and nutrition literacy [42]. Better food and nutrition literacy anticipated everyday intake of breakfast, lunch, and dinner compared to those who used to skip meals [43]. Participants having higher food- and nutrition-literacy scores reported lower consumption of sausages, hamburgers, and sweets [43]. Further, food and nutrition literacy were a barrier to dietary diversity and nutrient adequacy in school-age children in Iran [43]. A low level of functional food and nutrition literacy (FFNL) was significantly associated with a lower intake of fruits and meat products [43]. Likewise, inadequate food-label literacy predicted significantly lower consumption of dairy and meat products [43]. It was also found that poor food and nutrition literacy was significantly associated with a low level of nutrition adequacy ratio (NAR) of protein, calcium, vitamin B3, vitamin B6, and vitamin B9 and the probability of a lower-level mean adequacy ratio (MAR) [44]. Adolescents who were in contact with healthcare providers had better food- and nutrition-literacy scores [45]. Moreover, an increase in total nutrition literacy (T-NL), INL, and CNL enhance diet quality [49]. An increase in FNL was associated with lower sugar intake and better energy balance in male participants [49]. Further, higher education level was also a significant correlate with the nutrition literacy [48]. Nutritional literacy had a significant correlation with the field of study, residence, and body mass index (*p* < 0.05) [46]. Food and nutrition literacy and food insecurity have also been associated, and food-insecure participants were about three times more likely to have low food and nutrition literacy scores compared to the food-secure participants (OR = 2.86, 95% CI 1.35, 6.05; *p* = 0.006) [33].

The review findings of food literacy and nutrition literacy correlate with those reported by studies outside the MENA boundaries, with significant associations with a myriad of nutrition outcomes. Costarelli, V. et al. (2021) [67] debated that better parental nutrition literacy was significantly associated with the feeding practices of their children, including healthy-eating guidance and monitoring practices. Moreover, better food literacy was found to have a negative association with fast food consumption and alcohol abuse among secondary students (16–21 years old) in Portugal [68]. The relationship between food literacy and food security has been also addressed in Australia, showing that food-insecure participants were 1.4 times less likely to base their food choices on looking over the nutrition information panel [85]. Furthermore, food-label use was higher among Turkish adolescents having adequate nutrition literacy, who also experienced lower consumption of fast food and reliance on reliable sources to search for and access nutrition information [64], which were also emphasized by the findings reported by Ayer, Ç. and Ergin, A. (2021) [62] and Koca, B. and Arkan, G. (2020) [63]. Besides, there was a significant positive relationship between food literacy and students’ dietary behavior in Ghana [76]. Moreover, multiple studies reported a positive association between food literacy and adolescents’ dietary intake; adolescents with greater food knowledge and frequent food preparation behaviors had healthier dietary practices [13]. Taken together, improving communities’ food and nutrition literacy could refigure the nutrition situation in many countries, pushing them closer to or even attaining Goal 2 of the 2030 Agenda of Sustainable Development, which is to end all forms of malnutrition.

### 3.4. Existing Food Literacy and Nutrition Literacy Policies and Programs

The published literature reveals that the MENA countries have not adopted any food-literacy or nutrition-literacy policies and programs yet, despite the need and the evident effectiveness of the existing ones. On the other hand, many countries have taken the initiative to formulate and adopt school-based programs and policies to promote food and nutrition knowledge and skills among children, and in certain circumstances, those of their parents and other community members. According to Hawkes and colleagues [80], there are four mechanisms by which a policy can influence food preferences and support healthy-eating patterns: (1) policy provides an enabling environment for healthy-preference learning; (2) policy overcomes barriers to the expression of healthy preferences; (3) policy encourages people to reassess existing unhealthy preferences; and (4) policy stimulates a food-system response. Table 3 lists a collection of food-literacy and nutrition-literacy programs and policies, along with the countries in which they were implemented, the target group, a brief description of the program/policy, the theory they are based on, the effectiveness, and the evaluation tools. Countries such as Australia and the United States appear to have made the most efforts in developing effective programs to improve the food literacy and nutrition knowledge of intervened participants. Refer to Table 3 for more details about the existing policies and programs.

### 3.5. Limitations and Strengths of This Review

This review has some limitations. There is the potential for missing studies because only a few databases were searched. Nonetheless, the strength of this review is crystallized, contributing to the literature by highlighting the MENA’s undervaluation of the food-literacy and nutrition-literacy concepts in terms of original definitions, assessment, policies, and programs. Our findings can be used to inform researchers and policymakers to start taking incremental steps to improve the state of food literacy and nutrition literacy in the region.

## 4. Conclusions

Food and nutrition literacy is one of many factors contributing to the prevalence rates of malnutrition and health outcomes. This review emphasized that there is a current dearth of data, policies, and programs regarding food literacy and nutrition literacy in the MENA region. The authors present this review data to spur additional research efforts into these topics in such a region plagued by high rates of malnutrition and nutrition-related disorders. This review is a wake-up call for researchers and policymakers to develop a robust approach to address and combat food literacy and nutrition literacy concerns in the MENA region.

### Recommendations and Future Perspectives

The MENA countries should start taking steps to create a supportive environment that boosts the food literacy and nutrition literacy of people in the region. Nutrition education and food and nutrition programs and policies are indispensable to providing an enabling environment for healthy dietary behaviors and stimulating a food system response. The existing policies need to be evaluated and monitored formally to assess the extent of implementation and whether they are applicable in the MENA region, having the same promising short-term and long-term effective outcomes. Nutrition and food education in child care and school settings is one of many steps that can be initiated to pave the way to come near to achieving the sustainable development goals (SDGs) by 2030. Regional researchers should prioritize conducting sufficient studies on food and nutrition literacy as well, to serve as baseline data for stakeholders and policymakers for taking an action. It is the researchers’ responsibility to point out when and where to act, as it is the vulnerable population that benefits the most from intervention, and the best ways to develop, monitor, and evaluate food-literacy and nutrition-literacy programs.

## Figures and Tables

**Table 1:** Summary of definitions of food literacy and nutrition literacy over the years.

Author (Year)	Definition
Food Literacy	
Wickham, C. and Carbone, E. (2018) [21]	“Food literacy is about acquiring and developing the food-related skills necessary to help create behavior change”.
Truman, E. et al. (2017) [22]	“Food literacy involves broad sets of skills and knowledge about food origins and systems; individual and collective food experiences; food identification; physical, emotional and mental effects of food; as well as basic abilities related to food”.
Cullen, T. et al. (2015) [15]	“It’s the ability to make decisions to support the achievement of personal health and a sustainable food system considering environmental, social, economic, cultural, and political components”.
Vaitkeviciute, R., Ball, L., and Harris, N. (2014) [13]	“The scaffolding term that empowers individuals, households, communities or nations to protect diet quality through change and strengthen dietary resilience over time”.
Sustain Ontario (2013) [23]	“Food literacy involves understanding: where food comes from; the impacts of food on health, the environment and the economy; and how to grow, prepare, and prefer healthy, safe and nutritious food”.
Block, L. et al. (2011) [24]	“Whereas food knowledge is the possession of food-related information, food literacy entails both understanding nutrition information and acting on that knowledge in ways consistent with promoting nutrition goals and food well-being”.
Kolasa, K. et al. (2001) [25]	“The capacity of an individual to obtain, interpret and understand basic food and nutrition information and services and the competence to use that information and services in ways that are health-enhancing”.
Nutrition Literacy	
Lee, C.-K. et al. (2019) [26]	“Nutrition literacy concerns dietary performance, which reflects the competence of healthy-eating and has been shown to influence healthy-eating behaviors”.
Aihara, Y. and Minai, J. (2011) [19]	“Nutrition literacy may be defined as the degree to which people have the ability to obtain, process and understand basic diet information and the tools needed to make appropriate nutrition decisions”.
Silk, K. et al. (2008) [27]	“Nutrition literacy can be defined similarly to health literacy as the degree to which individuals can obtain, process, and understand the basic health (nutrition) information and services they need to make appropriate health (nutrition) decisions, with the qualification that the definition is nutrition specific”.

**Table 2:** Summary of studies assessing food and/or nutrition literacy in the MENA region.

Study Title	Author (s) (Year)	Country	Main Objective	Sample Size	Age	Assessment Tool	Nutrition/Food Literacy Level	Nutrition/Food Literacy Correlates
Nutrition Literacy among Adolescents and Its Association with Eating Habits and BMI in Tripoli, Lebanon	Taleb, S. and Itani, L. (2021) [39]	Lebanon	To investigate the association between nutrition literacy among adolescents and their BMI status and food habits.	*n* = 189 (57.4% males)	14–19 years old	NLAI	Adequate nutrition literacy on Nutrition and Health, Macronutrients, and food groups. Marginal nutrition literacy for household food measure, and food label reading.	Food habits score and Nutrition Literacy: adolescents with a lower food habits score had a higher Macronutrients literacy score. Adolescents’ BMI and their nutrition literacy: no associations.
Nutrition literacy is associated with income and place of residence but not with diet behavior and food security in the Palestinian society	Natour, L. et al. (2021) [40]	Palestine	To study the level of nutrition literacy among a group of Palestinians.	*n* = 101 (83.2% females)	>18 years old	NVS	About one quarter had adequate literacy scores.	Females, those with higher income, food label users and those who consumed low calorie products and checked health benefits on food label had higher literacy score. Literacy and eating habits: participants with adequate literacy reported lower use of high fat cheese, fried chicken and higher removal of chicken skin.
The relationship between Nutrition Literacy and Nutrition Information seeking attitudes and Healthy Eating Patterns in the Palestinian Society	Tell et al. (2021) [41]	Palestine	To describe FNL, INL, and CNL in the Palestinian society.	*n* = 149 (78% females)	>18 years old (mean age = 20.4 ± 4.9 years old)	Nutrition literacy scale	The mean of FNL was 2.8 ± 0.5 (over 7), INL was 3.3 ± 0.5 (over 8), and CNL was 3.6 ± 0.5 (over 11).	Nutrition literacy and food label use: FNL was correlated with the use of food label. CNL and INL were correlated with looking at ingredients part of the label. Nutrition literacy and food habits: NL correlated with the use of low fat, low calorie, serving size and health benefit., lower addition of cheese and mayonnaise to food. Nutrition literacy and source of nutrition information:
Food and nutrition literacy status and its correlates in Iranian senior high-school students	Ashoori, M. et al. (2021) [42]	Iran	To examine the food and nutrition literacy (FNL) status and its determinants in Iranian senior high-school students.	*n* = 755 (49.7% were females)	17–18 years old	FNLAT	The mean ± SD of the total food and nutrition literacy score was 52.1 ± 10.96 (below the minimum adequate level of 60).	Food and nutrition literacy and gender: girls had significantly higher functional score than boys, while food label score was significantly higher in boys as compared to girls. Food and nutrition literacy and school performance: high FNL score was significantly higher among students who had better school performance (OR = 1.13, CI = 1.06–1.20). Food and nutrition literacy and food label use: participants who had a family member with a nutrition-related disease had a higher score of food label reading skill (OR = 1.48, CI = 1.01–1.64).
Food and Nutrition Literacy (FNLIT) is Associated to Healthy Eating Behaviors in Children	Doustmohammadian, A. et al. (2021) [43]	Iran	To investigate associations between food and nutrition literacy (FNLIT) and eating behaviors of elementary school children in Tehran, Iran.	*n* = 803 (47.6% were females)	10–12 years old	FNLIT	68.8% included high-level cognitive domains. At least one out of four students (25%) included low FNLIT values in the skill domain.	Food and nutrition literacy and eating behaviors (meal patterns): High food and nutrition literacy scores in the cognitive domain were negatively associated to irregular breakfast intakes compared to everyday eating breakfast, irregular lunch intakes compared to everyday eating lunch and irregular dinner intakes compared to everyday eating dinner. Food and nutrition literacy and food habits: High food and nutrition literacy scores in the cognitive domain were attributed to never eating sausage/hamburger. Food and nutrition literacy scores in the skill domain were positively associated to never eating sweet snacks.
Relationship between household food insecurity and food and nutrition literacy among children of 9–12 years of age: a cross-sectional study in a city of Iran	Khorramrouz, F. et al. (2020) [33]	Iran	To assess the relationship between household food insecurity (HFI) with food and nutrition literacy (FNLIT) in a sample of Iranian children.	*n* = 315 (49% females)	9–12 years old	FNLIT	14% of the students had low FNLIT scores. Only 23.2% had high FNLIT scores.	Food and nutrition literacy and food insecurity: food-secure subjects had higher scores for total FNLIT (*p* < 0.001) and some subscales including understanding food and nutrition information (*p* = 0.01), nutritional health knowledge (*p* = 0.001), food choice literacy (*p* = 0.009) and food label literacy (*p* < 0.001). The food-insecure group had a higher likelihood of having low FNLIT compared to the food–secure group by about 3 times (OR = 2.86, 95% CI 1.35, 6.05; *p* = 0.006).
Low food and nutrition literacy (FNLIT): a barrier to dietary diversity and nutrient adequacy in school age children	Doustmohammadian, A. et al. (2020) [44]	Iran	To assess the relationship between Food and Nutrition Literacy (FNLIT) and dietary diversity score (DDS) and between FNLIT and nutrient adequacy (NAR%, MAR%) in school-age children in Iran.	*n* = 803 (52.1% boys)	10–12 years old	FNLIT	25% had low scores in skill domain (lower scores in critical food and nutrition literacy and food label literacy, while higher scores for food-choice literacy). The majority scored moderate to high in cognitive domain.	FNLIT and Dietary Intake Adequacy: Low levels of FNLIT were significantly associated with low level of nutrition adequacy ratio (NAR) of protein, calcium, vitamin B3, B6, B9, as well as the probability of lower level of mean adequacy ratio (MAR). FNILT and Dietary Diversity: Low FFNL was significantly associated with low DDS (lower fruit, meat and dairy diversity scores). Low level of food label literacy (FLL) was significantly associated with lower DDS (dairy and meat diversity scores)
Students’ Nutrition Literacy and the Existence of Health Care Providers in Iranian Schools	Mehri, A. et al. (2020) [45]	Iran	To assess the relationship between nutrition literacy and the existence of school health care in Iranian schools.	*n* = 504	13–15 years old	FNLIT	Most students had a low FNLIT (62% males and 58.1% females).	Nutrition literacy and being in contact with healthcare providers: The probability of low FNLIT was lower in students with health care providers than those without them (OR = 0.46, CI 95%; 0.10, 0.91).
Nutritional literacy status and its related factors in students of Yasuj University of Medical Sciences	Bahramfard, T. et al. (2020) [46]	Iran	To investigate the nutritional literacy among students at Yasuj University of Medical Sciences.	*n* = 397 (56.4% were females)	Mean age = 22.2 years old	EINLA	The mean score of students’ nutritional literacy was 24.9 out of 35. (1% of students had inadequate nutritional literacy and 50.9% and 48.12% of students had borderline nutritional literacy and adequate nutritional literacy, respectively).	Nutritional literacy was significantly correlated with the semester, field of study, students’ residence and body mass index (*p* < 0.05).
Food and nutrition literacy (FNLIT) and its predictors in primary schoolchildren in Iran	Doustmohammadian, A. et al. (2019) [47]	Iran	To describe the distribution of food and nutrition literacy (FNLIT) in a cross-sectional sample of 803 students aged 10–12 years from elementary schools in Tehran city, Iran.	*n* = 803	10–12 years old	FNLIT	The total FNLIT level was good. However, more than half of the children (69%) had high levels of FNLIT in the cognitive domain, but in the skills domain, very few (15%) scored highly.	FNLIT and sociodemographic variables: gender, parent’s education and age, and birth order.
Measuring Nutritional Literacy in Elementary School Teachers in Yasuj: A Cross-Sectional Study	Hemati, M. et al. (2018) [48]	Iran	To measure the nutritional literacy of primary school teachers in Yasuj.	*n* = 110	>18 years old	Nutrition Literacy Instrument developed in Turkey	Mean score of nutritional literacy was 27.14 ± 3.2, which indicated that 22.7% of teachers had inadequate nutritional literacy.	Teachers with fewer years of work and higher education had a higher level of nutritional literacy.
Nutrition literacy as a determinant for diet quality amongst young adolescents: A cross sectional study	Joulaie, H. et al. (2018) [49]	Iran	To assess the association between nutrition literacy and diet quality among young adolescents.	*n* = 388 (*n* = 249 females)	13–15 years old	NLQ-20: Nutrition Literacy Questionnaire	Total nutrition literacy (T-NL) mean and standard deviation was 52.98 ± 7.15.	Nutrition literacy and diet quality: Among boys, an increase in T-NL (OR: 1.049), INL (OR: 1.13), and CNL (OR: 1.086) enhance diet quality (increase in FNL was associated with lower sugar intake and better energy balance in boys).

**Table 3:** Policies and programs have been implemented to improve food and/or nutrition literacy.

Country of Implementation	Policy/Program Name	Target Group	Policy/Program Description and Objective	Theory	Effectiveness/Outcomes	Evaluation Tool (s)
MENA	None	No data	No data	No data	No data	No data
Australia	Oz Harvest’s primary-school Food Education and Sustainability Training (FEAST) program [86]	10–12 year-old students	A curriculum-aligned program in 20 primary schools, delivered as a 1.5-h lesson/week, for a 10-week unit of inquiry, incorporating theory and cooking. Designed to educate children about sustainability, food waste and nutrition, using hands-on cooking activities.	Precede-Proceed Planning model (PPM) and Social Cognitive Theory (SCT)	Primary outcomes: children’s self-reported fruits and vegetables intakes (serves/day). Secondary outcomes: Food literacy constructs such as: nutrition knowledge, food preparation and cooking skills, self-efficacy and behaviors, food waste knowledge and behaviors and food production knowledge.	A 25-item online survey was developed for administration at baseline (impact evaluation) and immediately post-intervention (impact and process evaluation).
Australia	OzHarvest’s NEST Program [87]	>18 year-old adults	A 6-week, 15 h guided public health nutrition program, integrating a series of nutrition activities, goal setting, and practical cooking lessons, utilizing recipes from OzHarvest’s Everyday (photo-based) Cookbook, and culminating in the sharing of a meal together. The objectives of the program are to: (1) improve participants’ food literacy, (2) increase consumption of core foods aligned with the Australian Dietary Guidelines, (3) decrease consumption of discretionary foods and drinks, (4) reduce household food insecurity, and (5) increase social engagement.	SCT	Improvements in food security status, cooking confidence, food preparation behaviors, nutrition knowledge, and average daily vegetable intake.	Pre–post surveys and follow-up interviews with NEST participants were conducted.
Australia	Food Sensations for Adults (FSA) [88]	>18 year-old adults	Consists of a series of four, two and a half hour sessions. Aims to improve food literacy by increasing skills in how to purchase and prepare healthy foods.	Health Belief Model and Social Learning Theory	Improvements in food literacy in 61–74% of program participants, manifested by an increase in self-reported fruit and vegetable intake, planning and management, selection, and food preparation.	14 items behavior checklist referred to as a food literacy behavior checklist and four short closed-ended questions on dietary behaviors.
Australia	Food Sensation for Schools (FSS) [89]	4–18 year-old students	A stand-alone 1- to 2-h session of hands-on nutrition education and cooking for students.	Social Learning Theory	Students develop positive attitudes towards healthy eating and knowledge about food and nutrition. The FSS sessions have improved knowledge and skills related to the dietary guidelines, food selection, food preparation, and safe food handling.	Quantitative and qualitative surveys.
Australia	Fuel Your Future (FYF) [90]	12–18 year-old youth	Four, 1- to 2-h stand-alone workshops to improve the cooking and food literacy of youth. Topics: Australian Guide to Healthy Eating, serve size vs portion size, fat sugar and salt investigation, food safety and storage, cooking.	Social Learning Theory and Socio Ecological Theory of behavior change.	Influence behavior change at individual, interpersonal, organizational and policy level by incorporating capacity building among students and health professionals.	Quantitative and qualitative surveys.
Australia	Food Sensation for Parents (FSP) [91]	Parents of children up to 5 years old	It is a free healthy eating and cooking program designed for parents. Consists of a series of five 2.5 h face-to-face OR four, 1.5 h online, fun, and interactive sessions that show parents how to choose and prepare healthy meals that are quick, delicious and low cost for their whole family.	Health Belief Model and Social Learning Theory	Parents learn: Healthy eating for the whole family/How to introduce solids and teach children to eat/Strategies to take the stress out of mealtimes/Lunchboxes, label reading and food safety/Budgeting and meal planning/Quick, easy, delicious, low-cost recipes.	Quantitative and qualitative surveys.
Australia	7-Week Food Literacy Cooking Program [92]	Adults >18 years old	The intervention group participants completed a cooking program consisting of weekly 90 min sessions for 7 weeks (new recipe each week) to increase cooking confidence.	NA	Significant post-program improvements in cooking confidence and satisfaction, ability to change eating habits, and overcome lifestyle barriers. Participation also improved mental and general health.	An online self-report questionnaire.
United States	Teens CAN: Comprehensive Food Literacy in Cooking, Agriculture, and Nutrition [93]	13–18 year-old teens	12 modules of experiential lessons and application activities within three topics (agriculture, nutrition, and cooking).	Social Cognitive Theory and Constructivism	Teens CAN provides a comprehensive and necessary approach to advancing food literacy in adolescents.	Overall confidence scores.
Italy	MaestraNatura Program [94]	6–13 year-old students	Active participation of students in experimental activities at school, with the involvement of parents in cooking activities.	NA	Increase food literacy and favor a healthier relationship with food. It is applicable in areas outside of Italy.	A pilot study carried out in nine Educational Institutes, a specific path was tested for effectiveness in increasing students’ knowledge about fruit and vegetables by conducting questionnaires before (T0) and after (T1) the didactic activities.
Canada	Cook IT UP! [95]	Mean age = 14.6 years old	A community-based cooking program for at-risk youth, focusing on food education and building cooking skills. Cooking Component: Twice monthly. Fieldtrip Component: Fieldtrips were selected to connect the youth to their cooking experiences. For example, a trip to a local sugar bush to learn how maple syrup was made complemented the cooking session on pancakes.	NA	Effective template for other agencies and researchers to utilize for enhancing existing programs or to create new applied cooking programs for vulnerable populations.	A pre/post cooking skills assessment questionnaire. Qualitative interviews were undertaken to determine the effectiveness of the program from the perspective of all participants involved.
Denmark	FOODcamp [96]	12 year-old students	Different food-related classes and activities for students. Schoolchildren’s FL has been defined in terms of five competencies: “to know” (food-related knowledge), “to do” (cooking skills), “to sense” (less well-explored sensory experiences), “to care” (taking care of oneself and others), and “to want” (willingness to take a stand and act).	NA	The program produces significant effects for the following FL dimensions: “to do”, “to sense” and “to know”, as well as for overall FL.	NA
Portugal	Health at the Table [97]	Children (6–10 years)	A food-literacy curriculum, consisting of weekly sessions of food literacy and nutrition education.	NA	Most of the teachers agreed that the curriculum was appropriate (69.2%) and that children developed health, wellness/well-being and environmental skills (83.1%). Most of the children had learned about healthy eating (86.3%) and claimed to eat healthier since the Health at the Table implementation (58.9%)	Weekly submission form into an online platform for each food-literacy session applied by the teacher.
United States	SNACC (Sustainable Nutrition and Community Connection) [98]	Youth	SNACC is a weekly after-school youth-development program that provides food to students and families in need and teaches middle- and high-school students to prepare healthy meals to enjoy with their families. SNACC engage youth with peers and chefs to try new foods and learn new skills. Students are invited back to hold leadership roles and help younger youth while still engaging in the weekly cooking sessions. Moreover, it provides meals and groceries for families.	NA	82% of students had started helping out more often in the kitchen, and learned how to eat healthier, while 90% of SNACC parents reported that their child was more confident in the kitchen.	NA
United States	Food Literacy Project’s Youth Community Agriculture Program (YCAP) [99]	Adolescents	Promote food system engagement. Employment opportunities to complete a harvest cycle, cook with local chefs, and develop entrepreneurial skills with local business owners.	NA	Helps teens to LEARN and EARN (passion for preparing and eating healthy food); Investigate community food justice; Conduct community nutrition education, outreach, and advocacy.	NA
United States	Food Literacy Partners Program [25]	Health professionals and community volunteers	The FLPP includes a training program and a commitment to provide volunteer service. The training program is a 20 h course focused on food and nutrition messages identified as important for the nutritional well-being of people in eastern North Carolina.	NA	Volunteers enhance their own knowledge and skills and enthusiastically share that knowledge and skills in service to their community.	NA

NA: Not Applicable.

## Data Availability

All the study data are reported in this paper.
